# Esophageal lymphangioma: a case report and review of literature

**DOI:** 10.1186/s12876-019-1026-9

**Published:** 2019-06-26

**Authors:** Yuqing Cheng, Xiaoli Zhou, Kequn Xu, Qin Huang

**Affiliations:** 1 Department of Pathology of the Affiliated Changzhou Second People’s Hospital of Nanjing Medical University, Changzhou, China; 2 Department of Oncology of the Affiliated Changzhou Second People’s Hospital of Nanjing Medical University, Changzhou, China; 3 Department of Pathology and Laboratory Medicine of Veterans Affairs Boston Healthcare System, Harvard Medical School and Brigham and Women’s Hospital, 1400 VFW Parkway, West Roxbury, MA 02132 USA

**Keywords:** Lymphangioma, Esophagus, Endoscopic submucosal dissection

## Abstract

**Background:**

Lymphangioma of the esophagus is an exceedingly rare benign tumor. Herein, we reported a case of lymphangioma in the thoracic esophagus.

**Case presentation:**

The patient was a 48-year-old woman who presented to our hospital with a one-month history of dysphagia. Upper endoscopy revealed an esophageal submucosal lesion that was completely removed by endoscopic submucosal dissection. Pathologic examination of the resected specimen secured the diagnosis of lymphangioma. A review of the PUBMED indexed literature in English with the key words of esophagus and lymphangioma was carried out and the results were discussed.

**Conclusion:**

Esophageal lymphangioma is a rare submucosal tumor and should be included in the differential diagnosis of esophageal submucosal tumors.

## Background

Lymphangioma is a benign microcystic lymphovascular lesion characterized by dilated lymphatic channels, and located primarily in the neck, axilla, and groin, as reported in the literature [1]. Lymphangioma rarely occurs in the esophagus [2]. The present report described a patient presented with dysphagia. Subsequent upper endoscopy discovered a broad-based, sessile, elevated submucosal esophageal lesion that was successfully resected endoscopically. Pathologic evaluation of the resected lesion disclosed the evidence for lymphangioma. In this case report, we analyzed the characteristics of white-light endoscopic appearances, endoscopic ultrasonography (EUS) signs, endoscopic resection, and histopathologic features of this benign tumor with a review of the relevant literature.

## Case presentation

**Fig. 1:**
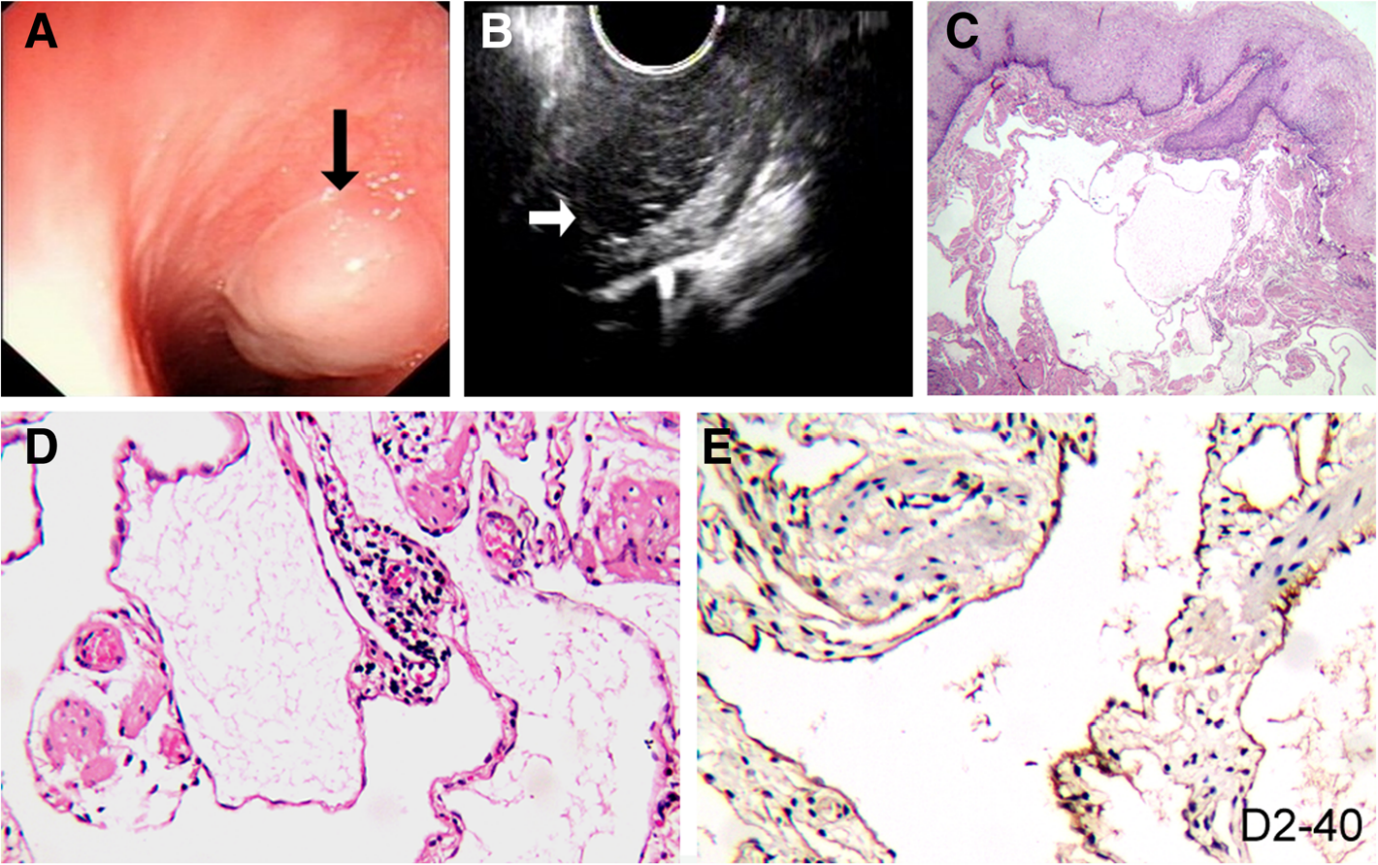
Endoscopic and histopathological findings of the esophageal lymphangioma. **a** sessile polypoid soft tumor with a lustrous surface was found endoscopically in the upper-mid esophagus at 20–22 cm from the incisor. **b** Under endoscopic ultrasonography, the tumor showed a heterogeneous hypoechoic pattern and involved the lamina propria and submucosal space, but not the muscularis propria. **c** Microscopically, the tumor was composed of dilated lymphatic vessels in various sizes underneath the normal esophageal squamous epithelium. **d** The lymphatic vessels were lined by flat benign endothelial cells with lymphocytic aggregates in the fibrous stroma. **e** Flat endothelial lining cells of this esophageal lymphangioma were immunoreactive to D2–40, a classical biomarker for lymphatic endothelial cells, confirming the diagnosis of lymphangioma

A 48-year-old woman complained of dysphagia for 1 month. In April 2018, she underwent esophagogastroduodenoscopy in our hospital and an esophageal submucosal tumor (SMT) was discovered in the upper-mid esophagus about 22–24 cm from the incisors. Under white light endoscopy, this lesion was broad-based, poorly defined, sessile, and elevated in size of 1.5 cm in diameter. The overlying mucosal surface was pale-whitish gray without ulcer or erosion (Fig. 1a). The adjacent esophageal mucosa was normal. There was no evidence of simon-red mucosal metaplastic changes. No additional tumor was identified. The stomach and duodenum were normal. Further endoscopic evaluation of this esophageal lesion with endoscopic ultrasonography (EUS) demonstrated a hypoechoic mass with heterogeneous echo and microcystic features; signs for blood flow were absent. The lesion was located primarily in the submucosal space without involvement of the underlying esophageal muscularis propria (Fig. 1b). This submucosal lesion was considered clinically as a benign lesion that was completely resected by endoscopic submucosal dissection (ESD) for histopathologic diagnosis and to relieve the patient’s symptoms.

The resected lesion measured 1.5 × 1.2 × 1.0 cm in size and exhibited whitish-gray, polypoid gross appearances. After routine formalin fixation, the lesion was serially sectioned to show whitish-gray, soft and vaguely spongy cut surface. No solid tumor or nodule was noted. No necrosis/hemorrhage was identified. Microscopically, the lesion involved both lamina propria and submucosa, but not muscularis propria, and was composed of thin-walled, micro-cystically dilated lymphatic channels in various sizes, which were separated by delicate fibrous stroma (Fig. 1c). The lymphatic channels were lined by flat endothelial cells with occasional small lymphocytic aggregates present between channels (Fig. 1d). Within some lymphatic channels was amorphous lymphoid fluid. Hemosiderin deposition and blood vessels invested by smooth-muscle layers were absent. No dysplasia or malignancy was identified in the tumor or the overlying squamous epithelium. By immunohistochemistry with valid controls, the lymphatic channel lining cells exhibited diffuse immunopositivity for D2–40 (Fig. 1e), but focal positivity for CD34, and negativity for FVIII.

The patient post-ESD hospital course was uneventful. She was well at a 12-month follow-up without complaints.

## Discussion and conclusions

Lymphangioma is a benign soft tissue tumor that rarely occurs in the gastrointestinal tract in adults [3]. About 1% of lymphangiomas were originated in the gastrointestinal tract, of which the most frequent location was the colon, followed by the stomach, duodenum, small intestine, and esophagus [2]. A PUBMED literature search identified only 30 cases of esophageal lymphangioma in English (Table 1). Although there is no evidence of increased incidence of this tumor, the detection of esophageal lymphangioma increased over the past decade with 14 cases reported, compared to merely 16 cases published over 70 years after its initial identification in 1934 [4], suggesting an increased use of upper endoscopy and awareness of endoscopists on esophageal lesions.

**Table 1:** Cases of Esophageal Lymphangioma in the English Literature

Case	Author	Year	Country	Gender	Age	Site	Size (cm)	Chief Complains	Treatment
1	Watson-williams [4]	1934	UK	Male	61	L	NA	Chest pain, vomiting	Observation
2	Schmidt [5]	1961	USA	NA	NA	NA	NA	NA	Autopsy
3	Brady [6]	1973	USA	Female	62	L	5	Epigastric pain	Observation
4	Armengol-Miro [7]	1979	Spain	Male	64	L	1	Epigastric pain	Snare polypectomy
5	Tmamada [8]	1980	Japan	Male	46	L	NA	Dysphagia, vomiting	Open surgery
6	Liebert [9]	1983	USA	Male	58	L	1.5	Dysphagia	Snare polypectomy
7	Castellanos [10]	1990	Spain	Female	66	M	2 × 1.5	Chest pain	Open surgery
8	Yoshida [11]	1994	Japan	Male	55	M	4	Heart burn	Open surgery
9	Suwa [12]	1996	Japan	Female	52	L	2.2 × 2	Dysphagia	Snare polypectomy
10	Scarpis [13]	1998	Italy	Male	64	L	1.5	Epigastric pain	Snare polypectomy
11	Lee [14]	2002	Korea	Male	37	M,L	NA	Dysphagia	INF α2a and partial polypectomy
12	Yoon [15]	2004	Korea	Male	72	L	5.1 × 2.3	Vomiting	Open surgery
13	Saers [16]	2005	Germany	Female	52	L	0.7	Dysphagia, chest pain, abdominal discomfort	Endoscopic mucosal resection
14	Sushil [17]	2007	USA	Male	68	L	1.4 × 1.4	Heart burn	Snare polypectomy
15	Best [18]	2008	USA	Male	68	U	3.5 × 2.2, 2 × 1.4	Dysphagia	CO2 laser resection
16	Seybt [19]	2008	USA	Male	53	L	4	Dysphagia, regurgitation	Open surgery
17	Arashiro [20]	2010	Japan	Female	33	L	1.9 × 1.5	Reflux symptoms	Endoscopic submucosal dissection
18	Lee [21]	2011	Korea	Male	55	M	0.7	NA	Endoscopic resection with band ligation
19	Xue [22]	2012	China	Male	58	M,L	10 × 2.5 × 1, 6 × 5 × 4	Dysphagia	Open surgery
20	Zhao [23]	2013	China	Male	35	U	0.8 × 0.6	Asymptomatic	Dual-channel endoscopic resection
21		2013	China	Female	42	M	0.8 × 0.5		
22		2013	China	Male	47	M	0.4 × 0.4		
23		2013	China	Male	38	M	1.2 × 0.7		
24		2013	China	Male	77	L	0.7 × 0.4		
25		2013	China	Female	50	M	0.8 × 0.5		
26	Barbosa [24]	2015	Portugal	Male	57	L	1.2	Asymptomatic	Observation
27	Luo [25]	2017	China	Male	41	L	6 × 1	Dysphagia	Endoscopic piecemeal mucosal resection
28	Zhao [26]	2017	China	Male	59	M	NA	Choking	Endoscopic mucosal resection
29	Hu [27]	2018	China	Male	46	M	16 × 6 × 4	Dysphagia	Endoscopic submucosal dissection
30	Min [28]	2018	China	Female	44	M	7	Dysphagia	Snare electrocautery
31	Present case	2019	China	Female	48	U	1.5 × 1.2 × 1	Dysphagia	Endoscopic submucosal dissection

UK: United Kingdom; USA: United States of America; L: Lower esophagus; M: Middle esophagus; U: upper esophagus; INF: interferon

Lymphangioma in the esophagus, unlike its counterpart in the gastrointestinal mesentery or head and neck region, tends to occur in adulthood, with the median and average age of 55 and 53.8 years (range 32–72), respectively. The male:female ratio is about 2.6 (21:8). In general, Esophageal lymphangioma is solitary in most reported cases (93.1%, 27/29), but multiple tumors (6.9%, 2/29) do occur. Although the tumor has a wide range in size, from 0.4 cm to 16 cm, the size of most tumors (74.1%, 20/27) is less than 5 cm with overall median and average sizes of 1.9 cm and 3.2 cm, respectively. The tumor is most frequently located in the distal esophagus (54.8%, 17/31). Interestingly, the clinicopathologic characteristics of this tumor differ in various ethnical patient groups. For instance, the patients’ median age is younger in Chinese than in non-Chinese (46 years in Chinese, versus 57.5 years in non-Chinese). In Chinese patients, esophageal lymphangioma shows a predilection of upper- and middle-esophagus location (75%, 9/12), whereas only 5 of 19 tumors (26.3%) located in the same site in non-Chinese patients. Moreover, the tumor size is also larger in Chinese patients. Among the patients with tumor size exceeding 5 cm, 80% (4/5) are Chinese. Understandably, the increased detection of this rare tumor in China may be related to the widespread availability of upper endoscopy among ordinary citizens in this most populous country in the world.

Overall, clinical presentations of patients with esophageal lymphangioma are nonspecific. They may be asymptomatic or may have various chief complaints, depending upon the location and size of a tumor. Dysphagia, as shown in our case, is the most common [14, 25, 27, 28]. Other common symptoms include heartburn and epigastric pain, which may be related to coexisting gastropathy and reflux disease [13, 22]. There are still some tumors identified incidentally [18, 23].

Endoscopically the tumor is pale-pink, whitish gray, or watery yellowish, polypoid, and translucent; it is pliable when compressed by the biopsy forceps [13, 23–25]. The overlying mucosa is normal-appearing under white light endoscopy. A large tumor may be lustrous and translucent [27, 28]. Esophageal EUS is routinely used to evaluate the size and depth of a lesion. The classical characteristics of esophageal lymphangioma under EUS manifest a honeycomb- or grid-like multi-microcystic echo pattern and the lesion may involve lamina propria and submucosal layer. Sometimes, the echo pattern may vary, according to the size of dilated lymphatic vessels [17, 20, 27, 28]. EUS examination is very helpful to differentiate lymphangioma from leiomyoma, the most frequent esophageal SMT, because EUS is able to clearly exhibit the micro-cystic echo pattern and the underlying intact muscularis propria.

Microscopically, esophageal lymphangioma is characterized by localized proliferation of thin-walled, dilated lymphatic channels in various sizes, as shown in the current case. There is no dysplasia in the overlying squamous epithelium, except in 1 case reported by Scarpis et al*,* who described focal low-medium grade dysplasia in the squamous epithelium overlying the tumor [13]. In most cases, it is not difficult to establish the correct diagnosis based on histological features. In the cases needed to be differentiated from hemangioma, the diagnosis of lymphangioma can be confirmed by a positive immunostaining pattern in lymphatic endothelial cells for D2–40 and a negative immunoreactivity for FVIII, while the expression of CD34 is variable [1].

Different treatment modalities may be used for esophageal lymphangioma, according to the tumor size. Since the absence of published reports on malignant transformation of lymphangioma, the patient with a confirmed diagnosis of esophageal lymphangioma can be managed conservatively. A large symptomatic tumor may be resected surgically. Previously, endoscopic therapy was not used to resect esophageal lymphangioma in size of larger than 2 cm [17]. Nowadays, with the improvement in endoscopic methods and accumulating operative experience by endoscopists, large tumors in size up to 16 cm have been reported to be completely and successfully removed endoscopically without major adverse outcomes [25]. Endoscopic resection has become the treatment of choice for gastrointestinal SMT with advantages over surgery in safety, effectiveness, minimal injury, and better quality of life after resection. Numerous endoscopic treatment methods have been gradually used to resect esophageal lymphangioma, such as dual-channel endoscopic resection, endoscopic resection with ligation device, cap-assisted endoscopic mucosal resection, endoscopic mucosal resection with an electrocautery snare, laser resection and ESD [16, 18, 23, 27, 28].

In conclusion, esophageal lymphangioma is a rare submucosal tumor and should be included in the differential diagnosis of esophageal SMT. EUS plays an important role in preoperative diagnosis and evaluation of the tumor size and depth. At present, endoscopic resection appears to be the treatment of choice for suitable patients to relieve symptoms and render a definitive histopathologic diagnosis. Histopathologic evaluation demonstrates characteristic proliferation of variably-sized lymphatic channels with auxiliary immunostaining patterns for D2–40, FVIII, and CD34.

## Data Availability

All the data regarding the findings are available within the manuscript.

## Abbreviations

ESD: endoscopic submucosal dissection
EUS: endoscopic ultrasonography
SMT: submucosal tumor
